# Reconsidering indoor radon monitoring strategies in Romanian medical buildings: a data-driven perspective

**DOI:** 10.3389/fpubh.2026.1959204

**Published:** 2026-09-25

**Authors:** Ancuţa Ţenter, Tiberius Dicu, Gabriel Dobrei, Ştefan Florică, Mircea Claudiu Moldovan, Alexandru Lupulescu, Andrei Ceclan, Alexandra Cucoş

**Affiliations:** 1 Faculty of Environmental Science and Engineering, “Constantin Cosma” Radon Laboratory (LiRaCC), “Babeş-Bolyai” University, Cluj-Napoca, Romania; 2 Faculty of Electrical Engineering, Technical University of Cluj-Napoca, Cluj-Napoca, Romania

**Keywords:** healthcare facilities, medical buildings, radon monitoring, Romania, seasonal correction factors

## Abstract

**Introduction:**

Medical buildings are complex indoor environments with heterogeneous room functions, occupancy patterns and HVAC (especially ventilation) requirements. Although radon monitoring methodologies are applied across workplaces, healthcare facilities differ from residential buildings in air exchange, operational schedules and functional zoning. This study investigated indoor radon concentration in Romanian medical buildings and assessed whether current monitoring assumptions, including residential-derived seasonal correction factors, adequately reflect observed exposure patterns.

**Materials and methods:**

A total of 1700 CR-39 detectors were installed for three-month measurements in 115 medical institutions from 40 localities across Romania. After retrieval, 1,651 valid measurements were included in the final analysis. Annual indoor radon activity concentration (AIRAC) was estimated using seasonal correction factors recommended by the Romanian methodology. Mixed-effects models were fitted separately for log-transformed uncorrected three-month radon concentrations and seasonally corrected AIRAC, with floor level, facility type and starting month as fixed effects and building code as a random intercept.

**Results:**

Overall radon levels were low, with an arithmetic mean of 61 Bq/m^3^, a median of 40 Bq/m^3^ and a geometric mean of 41 Bq/m^3^. Only 26 of the 1,651 valid measurements, representing 1.6%, exceeded the reference level of 300 Bq/m^3^. Exceedances were concentrated on lower levels: 25 occurred in basements, semi-basements or ground-floor rooms, whereas only one was found on upper floors. Higher median concentrations were observed in radiology clinics and dental offices, while hospitals and medical clinics showed lower values. For seasonally corrected AIRAC, the mixed-effects model indicated significant effects of floor level, facility type and starting month. However, starting month was not significant in the parallel model fitted to uncorrected three-month concentrations.

**Discussion:**

Radon variability in medical buildings appears shaped by floor level, facility type and building-specific characteristics. The comparison between corrected and uncorrected seasonal patterns showed that the application of residential seasonal correction factors was associated with an amplified warm–cold contrast and a stronger starting-month pattern in AIRAC estimates.

**Conclusion:**

This first large-scale assessment of indoor radon in Romanian medical buildings supports a risk-based and context-sensitive monitoring strategy. Detector allocation should prioritize lower levels and facility categories associated with higher concentrations, while the use of residential seasonal correction factors in medical buildings should be further evaluated through repeated seasonal measurements.

## 1 Introduction

Hospitals and other healthcare facilities are complex indoor environments whose primary role is to support diagnosis, treatment, recovery and care (1, 2). Unlike many other public buildings, medical facilities accommodate multiple categories of occupants with different exposure patterns, such as medical staff with a 6 h/8 h/12 h work schedule and 24 h guards both day and night, administrative/technical staff with an 8 h work schedule during the day, patients of all ages, whose time spent indoors depends on their clinical condition and duration of care, and last but not least, the patients’ relatives who accompany them. Indoor air quality (IAQ) in such buildings is therefore directly connected to both patient safety and occupational health (1–3). Medical buildings comprise a range of functionally distinct spaces, including patient rooms, operating theatres, intensive care units, radiology, CT, MRI departments, laboratories, treatment rooms, administrative offices, technical and storage spaces, corridors and waiting spaces, and basement areas. Such functional heterogeneity has been associated with substantial differences in occupancy, ventilation requirements and contamination risk (2, 4–6). In addition, hospital activities themselves may contribute to indoor air contamination through cleaning and disinfection procedures, sterilization processes, anesthetic gases, pharmaceutical and laboratory chemicals, medical equipment and high occupant density (1, 4, 6–8). Consequently, IAQ in healthcare facilities is shaped by a combination of building design and operation (2), ventilation systems and maintenance practices (1, 5, 6), medical and occupant activities (4, 9), human presence (1, 2, 6), and outdoor air contributions (6, 10, 11). In medical facilities, the design and operation of heating, ventilation and air-conditioning (HVAC) systems are particularly important, as ventilation source, air-change rate, air movement, relative humidity, CO_2_ level, air filtering, temperature and occupancy have been associated with the diversity and composition of airborne bacterial communities, as well as with airborne microbial loads and the transmission of infectious agents (12–15). Mechanically ventilated patient rooms were reported to contain less diverse airborne bacterial communities than window-ventilated rooms, while controlled indoor thermal conditions have been associated with patient recovery and other medical outcomes (1, 13, 16–18). Conversely, inadequate HVAC design or operation, insufficient coverage of critical areas, or disruption of intended airflow patterns by personnel, equipment and door openings may compromise contamination-control objectives by reducing the removal of bacteria-carrying particles from the sterile field and incision zone, with potential implications for surgical wound contamination (19–23). However, ventilation requirements in hospitals are not uniform, but depend on the function and risk profile of each space. This functional heterogeneity is reflected in regulations and technical standards defining distinct requirements for air renewal, air-change rates, filtration and pressure control across different functional areas (24, 25). Thus, from an IAQ and building-operation perspective, medical facilities differ substantially from residential buildings and many other workplaces. Despite the growing interest in IAQ in healthcare facilities, radon remains rarely integrated into indoor environmental assessments of medical buildings, even though the World Health Organization recognizes it as one of the most important indoor air pollutants affecting human health (26). Recent reviews on IAQ in healthcare units show that most studies focus on parameters such as carbon dioxide, particulate matter, temperature, relative humidity, total volatile organic compounds, formaldehyde, carbon monoxide, nitrogen dioxide and microbiological contamination, whereas radon is usually addressed separately or only as an isolated parameter (1, 6, 7, 11). This gap is relevant because radon is an indoor pollutant whose concentration is strongly influenced by building characteristics, soil contact, floor level, pressure differences, ventilation regime and building operation patterns (27–30). Occupancy duration and work schedules are relevant primarily for exposure assessment, as they determine the time spent by different categories of occupants in indoor environments where radon may accumulate.

Previous studies in hospitals and healthcare-related buildings have shown that radon concentrations are often higher in basements, semi-basements and ground-floor areas, but also that localized high values may occur even in buildings where average concentrations are relatively low (31–36). Moreover, studies performed in hospital basements and air-conditioned medical rooms suggest that ventilation or lack of it and air-conditioning operation may substantially influence radon accumulation, sometimes more strongly than local geological characteristics alone (28, 32). These findings indicate that radon monitoring in medical buildings should account not only for geogenic radon potential, but also for floor level, room function and ventilation practice.

In Romania, the national methodology for determining radon concentration in residential buildings and workplaces provides general requirements for radon monitoring, including the use of integrated measurements, a minimum measurement duration of three months and a preference for measurements performed during the cold season (37). When measurements are shorter than one year, annual indoor radon activity concentration (AIRAC) is estimated by applying seasonal correction factors. However, these correction factors were derived from residential buildings, typically without considering the presence, type or operating schedule of mechanical ventilation systems. A similar issue concerns detector allocation within buildings. The number of detectors is set according to building type, floor area, and storey: for buildings with high occupancy over several floors, such as medical and educational facilities, the methodology prescribes at least one detector per homogeneous zone, one per 150 m^2^, and at least one additional detector per 250 m^2^ on each upper floor (37). While this approach ensures broad spatial coverage, it may not fully reflect the observed distribution of radon risk in complex medical buildings, where radon accumulation is expected to be more likely on basement, semi-basement and ground-floor levels, as well as in poorly ventilated spaces. Therefore, field data are needed to assess whether current monitoring strategies can be optimized without compromising risk detection.

As such, the present study investigates indoor radon concentrations in 115 Romanian medical institutions, including hospitals, medical clinics, dental offices, radiology clinics, veterinary offices and other healthcare-related facilities. The objectives were: (i) to characterize indoor radon levels in Romanian medical buildings; (ii) to assess the influence of floor level, facility type, room function and measurement starting month on radon concentrations; (iii) to evaluate the distribution of reference-level exceedances across floor levels and facility types; and (iv) to evaluate how the application of residential seasonal correction factors affects AIRAC estimates and starting-month patterns in medical buildings.

## 2 Materials and methods

### 2.1 Study area and radon measurements

The dataset used in this study was obtained within the framework of regulatory indoor radon monitoring performed in medical facilities. The monitored facilities were therefore not selected using a random sampling design, and the sample should not be interpreted as nationally representative. A total of 1700 CR-39 nuclear track detectors were placed in 115 medical institutions/facilities. The term “institution” refers to the monitored healthcare entity; in some cases, particularly hospitals, one institution could include more than one physical building. Of these, 40% were hospitals, 34% dental offices, and 9% medical clinics, with a similar percentage corresponding to veterinary practices. In addition, measurements were also conducted in seven radiology offices, two general medical offices, and one sanatorium. In terms of spatial distribution, measurements were carried out in 40 localities across 10 Romanian counties, with the majority conducted in Cluj County (48%), followed by Bihor (16%) and Alba (15%) counties (Figure 1). Rural areas are represented in the current study, accounting for 28% of the investigated localities.

**Figure 1:**
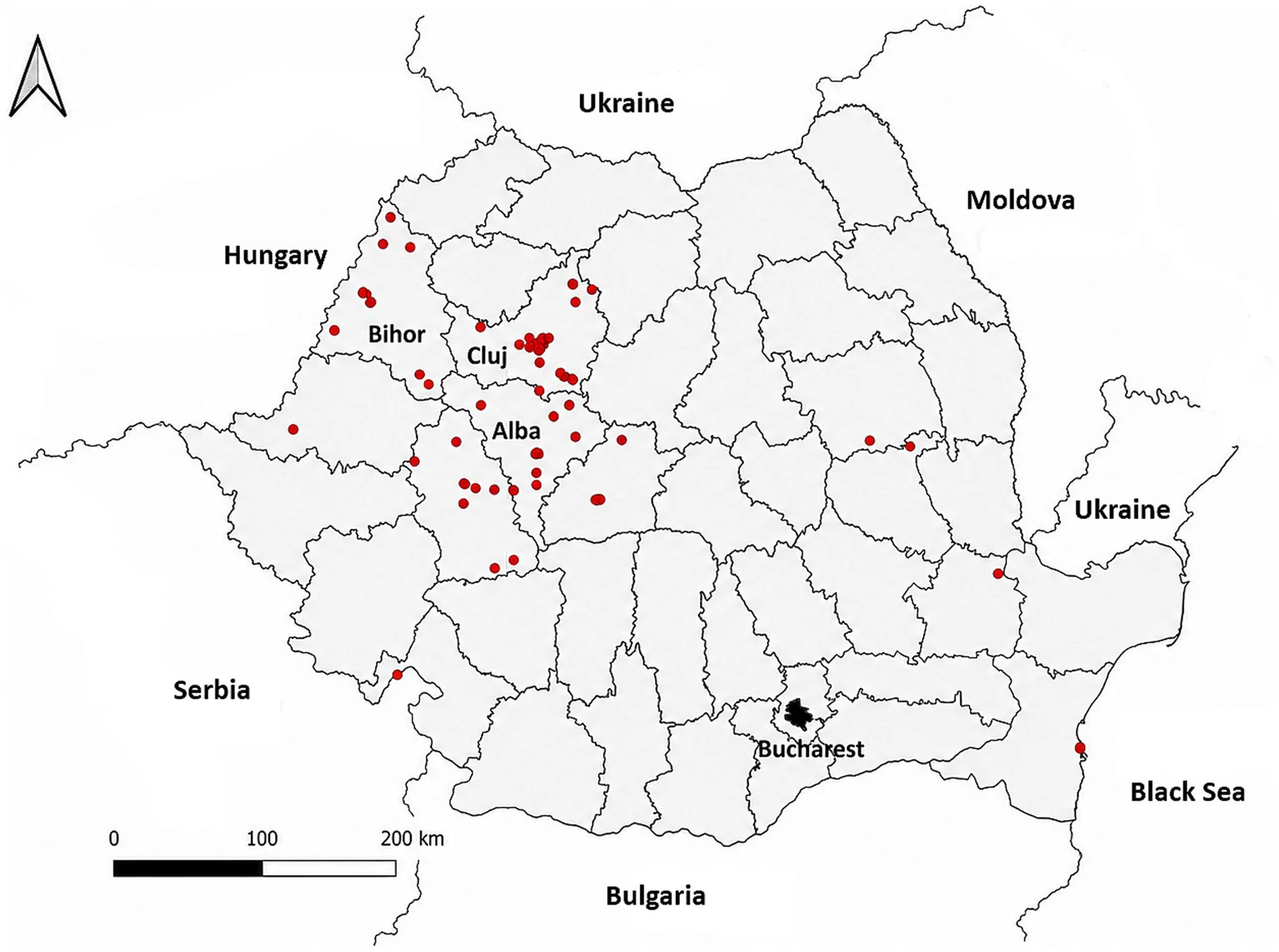
Spatial distribution of the surveyed medical institutions across Romania, including 1700 radon measurements performed in 115 institutions from 40 localities.

The detectors were placed for a monitoring period of three months. Although Romanian legislation explicitly recommends that such measurements be conducted during the cold season, operational constraints required measurements to be initiated throughout the year. As a result, detectors were placed during all months of the year, with an average monthly placement rate of 8%. Nevertheless, monthly variations were observed, ranging from a minimum of 4% (in April and September) to a maximum of 13 and 14% in March and January, respectively. For the seasonal analysis, the cold season was defined as the heating-dominated period from November to March, while the warm season was defined as the non-heating period from May to September. April and October were considered transitional months because heating operation, window-opening behavior and ventilation patterns may vary substantially during these periods.

Following the monitoring period, 1,651 detectors were successfully retrieved, resulting in a loss rate of approximately 3%. Subsequently, the detectors were processed at the “Constantin Cosma” Radon Testing Laboratory (LiRaCC) using equipment provided by Radosys (Hungary), following the protocol detailed by Cucoş et al. (38). The accuracy of the measurement system is verified annually both by the manufacturer and through participation in the international intercomparison exercise organized by the German Federal Office for Radiation Protection (Bundesamt für Strahlenschutz, BfS). The relative combined standard uncertainty associated with radon measurements was concentration-dependent, being approximately 25% for concentrations close to the background level and below 10% for concentrations above 100 Bq/m^3^.

Given the three-month exposure duration, the measured radon concentrations were adjusted using seasonal correction factors to estimate the annual indoor radon activity concentration (AIRAC), according to Equation 1:

$$\text{AIRAC}=C_{m}\times F_{c}$$ (1)

where: C_m_ is the measured radon concentration over the three-month exposure period and Fc is the seasonal correction factor. For each measurement, Fc was selected from the Romanian methodology as the value corresponding to the month in which the measurement started and to the three-month exposure duration. These factors are defined in the national methodology for indoor radon assessment, approved by the Romanian regulatory authority through Order No. 153/2023 (37). Notably, the correction factors were derived from measurements conducted in residential buildings, without accounting for the type of ventilation system.

### 2.2 Data analysis

Statistical analyses were performed using IBM SPSS Statistics 24 (IBM Corp., Armonk, NY, USA) and OriginPro 2024 (OriginLab Corporation, Northampton, MA, USA). Analyses were performed at two levels: building level and measurement level. Building-level AIRAC was calculated as the arithmetic mean of all valid measurements within each medical institution and was used for comparisons between facility types. Measurement-level data were used for analyses according to floor level, room function and starting month. The Kruskal–Wallis test was used for comparisons involving three or more groups, followed by Dunn’s *post hoc* comparisons with Holm adjustment for multiple testing. The Pearson’s correlation coefficient and Spearman’s rank correlation coefficient were calculated between the arithmetic mean AIRAC for each room-function category and the minimum fresh air supply rate per room volume specified in NP 015–2022. Only room-function categories for which a regulatory air-supply value was available were included in this analysis. In addition, a partial Pearson correlation was calculated between the same two variables while controlling for the percentage of measurements performed on upper floors within each room-function category.

To account for the hierarchical structure of the dataset, a linear mixed-effects model was fitted. To distinguish between measured radon variability and the effect introduced by seasonal correction, the model was fitted separately to two dependent variables: log-transformed uncorrected three-month radon concentration and log-transformed seasonally corrected AIRAC. Floor level, type of medical facility and starting month of measurement were included as fixed effects, while building code was included as a random intercept to account for the clustering of measurements within the same building. As a sensitivity analysis, the mixed-effects model was refitted by adding locality code as an additional random intercept, used as an indirect proxy for locality-level geographical and potential geogenic variability. The significance level α was set at 0.05.

## 3 Results

Among the 115 medical institutions investigated in the present study, 51 (44%) had fewer than five detectors installed per building, while at the opposite end, approximately 12% of the buildings were equipped with more than 30 detectors each. The median number of detectors placed per building was 5. In the case of hospital buildings, the median number of detectors installed was 21, with values ranging from 2 to 109. This heterogeneous distribution is explained by the type and surface area of the monitored medical institutions.

Table 1 presents the descriptive statistics of building-specific mean AIRAC values according to medical facility type. These values ranged from 16 to 1279 Bq/m^3^, with the maximum recorded in a dental office. This value (1279 Bq/m^3^) represents the highest mean AIRAC calculated for a single investigated medical building. In terms of median building-specific mean AIRAC, the highest values were observed in radiology clinics (138 Bq/m^3^), followed by medical offices (129 Bq/m^3^), dental offices (93 Bq/m^3^), veterinary offices (79 Bq/m^3^), hospitals (55 Bq/m^3^), medical clinics (47 Bq/m^3^) and the single sanatorium (27 Bq/m^3^). However, the medical office and sanatorium categories included only two and one institutions, respectively, and were therefore retained for descriptive purposes only. After excluding these two categories, the Kruskal–Wallis test indicated significant differences in the distribution of building-specific mean AIRAC values among facility types. Dunn’s pairwise *post hoc* comparisons with Holm adjustment showed that building-specific mean AIRAC values were significantly higher in dental offices than in hospitals (Holm-adjusted *p* = 0.008). Other pairwise comparisons were not statistically significant after adjustment for multiple testing.

**Table 1:** Descriptive statistics of building-level mean AIRAC values (Bq/m^3^), grouped by type of medical institution.

Medical institution	N*	Min	AM	Mdn	IQR	Max
Hospital	46	16	62	55	41	166
Dental office	39	18	135	93	97	1279
Medical office	2	99	129	129	—	158
Medical clinic	10	24	58	47	54	131
Radiology clinic	7	28	132	138	98	340
Sanatorium	1	27	27	27	—	27
Veterinary office	10	21	105	79	93	283

^*^N, number of medical institutions; Min, minimum; AM, arithmetic mean; Mdn, median; IQR, interquartile range; Max, maximum.

A high degree of variability was observed among the radon concentrations measured within the same building. For hospital-type buildings, the mean coefficient of variation (CV) was 78%, with values ranging from 5 to 173%. The highest variability was recorded in a building in which monitored rooms were distributed across multiple floors and served different functional purposes. Therefore, subsequent analyses were performed by floor level and room function.

Table 2 presents the descriptive statistics for AIRAC by the floor level at which the measurement was conducted. As expected, higher values, in terms of the mean, median, and maximum measured radon concentrations, were observed on the lower floors (basement, semi-basement, and ground floor). As such, median AIRAC values ranged from 45 Bq/m^3^ in semi-basements to 51 Bq/m^3^ in basements, whereas on the upper floors, median values ranged from 22 Bq/m^3^ on the third floor to 31 Bq/m^3^ on the first and fifth floors. A statistically significant difference was found between these lower floors and the upper floors using the Kruskal-Wallis test (*p* < 0.05). However, when the analysis was limited to the basement, semi-basement, and ground floor, no statistically significant differences were identified using the same non-parametric test. A similar result was obtained when applying the Kruskal-Wallis test exclusively to the upper floors (levels 1 to 7), with no significant differences found between AIRAC medians. The higher variability of AIRAC observed on lower building levels may reflect the greater proximity to soil as the primary radon source, differences in room function and the unequal distribution of measurements across floor levels (Figure 2).

**Table 2:** Descriptive statistics of AIRAC (Bq/m^3^), grouped by floor level.

Floor level	N*	Min	AM	Mdn	Max	No. (%) > RL
Basement	81	9	98	51	1733	3 (3.7)
Semi-basement	200	7	67	45	767	4 (2.0)
Ground floor	856	6	71	48	825	18 (2.1)
1	316	6	42	31	597	1 (0.3)
2	104	6	31	26	149	—
3	34	6	25	22	87	—
4	23	7	26	24	61	—
5	11	12	33	31	59	—
6	21	7	33	23	102	—
7	5	8	26	28	42	—

^*^N, number of measurements; Min, minimum; AM, arithmetic mean; Mdn, median; Max, maximum; No. (%) > RL, number (percentage) of measurements exceeding the reference level of 300 Bq/m^3^.

**Figure 2:**
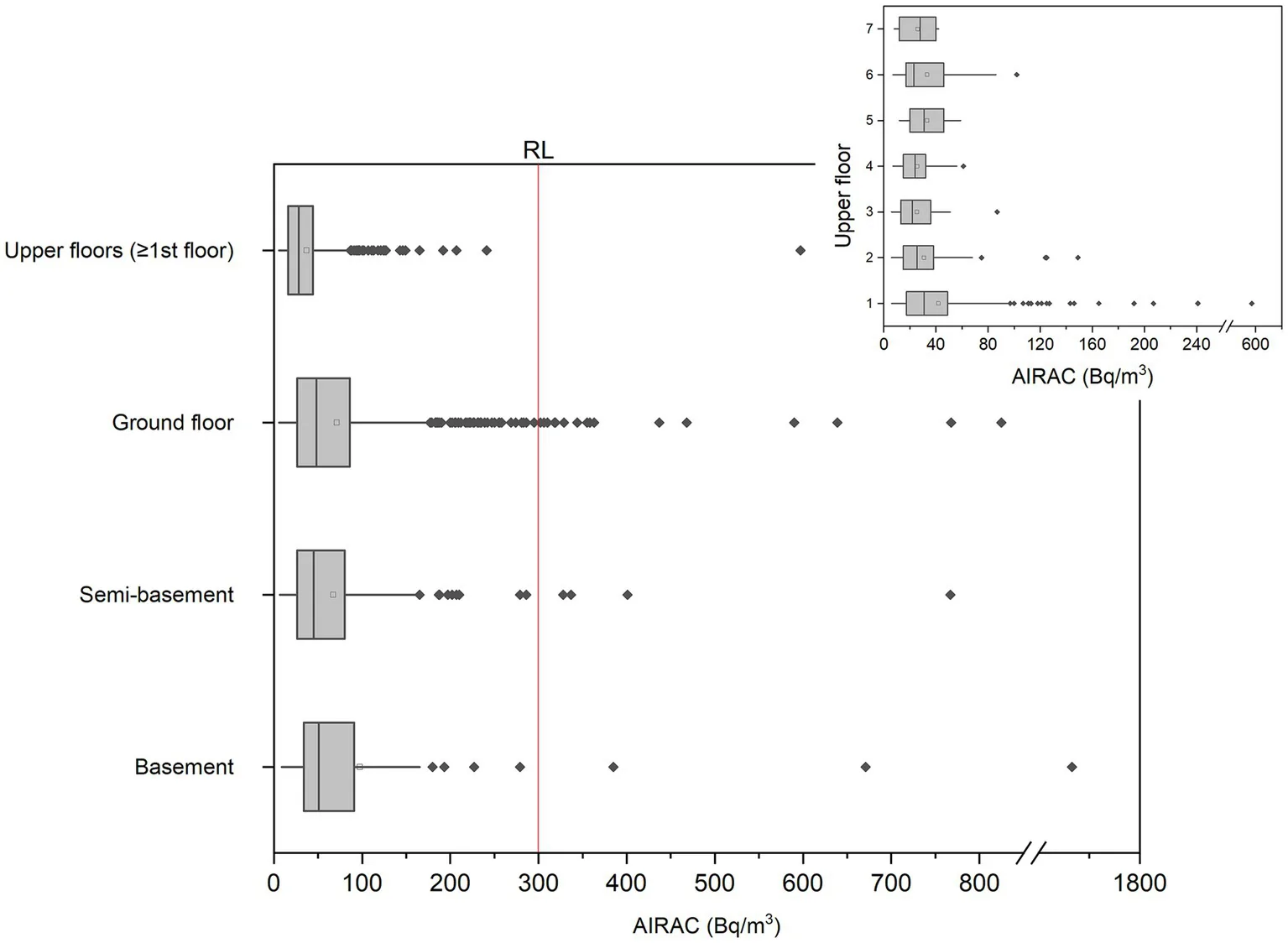
AIRAC variation with floor level. The inset illustrates data from upper floors (1st to 7th level). The red vertical line marks the reference level of 300 Bq/m^3^.

Of the 1,651 valid measurements, 26 (1.6%) exceeded the reference level of 300 Bq/m^3^. Exceedances were strongly concentrated on lower building levels. Specifically, 25 of the 1137 measurements performed in basements (3), semi-basements (4) and ground-floor rooms (18) exceeded the reference level, corresponding to an exceedance rate of 2.2%. By contrast, only 1 of the 514 measurements performed on upper floors, defined as the first floor or higher, exceeded the reference level, corresponding to an exceedance rate of 0.2%. These 26 room-level exceedances were distributed across 13 monitored medical buildings, corresponding to 11.3% of the investigated facilities. The number of exceedances per affected building ranged from 1 to 5. The 13 buildings with at least one exceedance included 8 hospitals, 3 dental offices, 1 radiology clinic and 1 veterinary office. At measurement level, the 26 exceedances comprised 17 measurements in hospitals, 4 in dental offices, 4 in radiology clinics and 1 in a veterinary office.

From the perspective of room function (Table 3), approximately one quarter of the measurements were conducted in treatment rooms, followed by administrative offices (around 20%), medical offices (17%), and radiology units (12%). Other areas included laboratories, dental clinics, patient lounges, pharmacies, operating theatres, veterinary offices, and kitchens. The category “Other” comprised spaces such as workshops, central heating rooms, changing rooms, doormen’s areas, ironing rooms, drying rooms, hallways, and similar utility spaces. Table 3 also includes the minimum prescribed air-change rates for the corresponding room categories, as specified in the Technical Norm for Hospital Buildings – NP 015–2022, approved by the Romanian Ministry of Development, Public Works and Administration (24). These values represent regulatory requirements rather than actual ventilation rates measured during the radon-monitoring period.

**Table 3:** Descriptive statistics of AIRAC (Bq/m^3^), grouped by type of room across medical building.

Room function	N*	Min (Bq/m^3^)	AM (Bq/m^3^)	Mdn (Bq/m^3^)	Max (Bq/m^3^)	CV (%)	Min. fresh air supply rate per room volume (1/h)^#^
Operating theatre	28	9	28	27	64	62%	20
Medical office	285	6	42	27	590	125%	6
Treatment room	380	6	48	36	401	97%	5
Kitchen	62	8	50	34	468	126%	2
Patient lounges	42	6	51	19	329	136%	3
Laboratory	90	6	54	37	385	111%	6
Pharmacy	42	10	69	53	222	79%	4
Administrative	318	6	71	44	768	130%	6
Other	148	6	75	52	671	103%	5
Radiology	203	7	85	59	1733	159%	5
Veterinary office	9	39	101	82	283	71%	-
Dental office	44	28	111	74	825	115%	-

^*^N, number of measurements; Min, minimum; AM, arithmetic mean; Mdn, median; Max, maximum; CV, coefficient of variance; ^#^The recommended minimum fresh air supply rate per unit room volume, expressed as 1/h, as specified in NP 015–2022.

The low values observed for both the mean and median AIRAC in operating theatres, medical offices and patient lounges may partly reflect the fact that more than half of the measurements — approximately 80% in operating theatres — were conducted on upper floors. Evaluating the relationship between AIRAC and the minimum fresh air supply rate, as recommended by Romanian technical norms, is an intuitively appealing approach. Pearson’s correlation coefficient indicated a moderate but non-significant negative association between arithmetic mean AIRAC and minimum fresh air supply rate (r = −0.54, *p* = 0.11). Spearman’s rank correlation for the same variables was weaker and also non-significant (r_S_ = −0.24, *p* = 0.50). However, when controlling for the percentage of measurements performed on upper floors within each room-function category, the partial correlation became much weaker (r = 0.10). This suggests that the apparent association between AIRAC and regulatory air-supply requirements was strongly influenced by the floor distribution of the monitored rooms.

The linear mixed-effects model fitted to seasonally corrected AIRAC confirmed that floor level, medical facility type and starting month were significantly associated with log-transformed AIRAC. Floor level showed the strongest effect (*p* < 0.001), followed by medical facility type (*p* < 0.001) and starting month (*p* = 0.046). To account for potential locality-level geographical or geogenic variability, locality code was included as an additional random intercept. Based on variance partitioning, locality accounted for approximately 2% of the total unexplained variance in log-transformed AIRAC, whereas differences between buildings accounted for approximately 36%. These results suggest that the observed facility-type effect was not explained primarily by locality-level variability. Estimated marginal means indicated that, after adjustment for facility type, starting month and within-building clustering, radon concentrations remained highest in basements and semi-basements. Back-transformed model-estimated concentrations were approximately 95 Bq/m^3^ for basements, 82 Bq/m^3^ for semi-basements and 64 Bq/m^3^ for ground-floor rooms, compared with approximately 35–46 Bq/m^3^ on upper floors. Adjusted estimates were higher for dental offices and radiology clinics than for hospitals and medical clinics, supporting the descriptive finding that these facility types showed higher radon concentrations.

The highest adjusted AIRAC values were observed for measurements initiated in June and July, suggesting that residential seasonal correction factors may amplify month-related differences when applied to medical buildings. The Kruskal–Wallis test showed significant differences according to the starting month of measurement for both corrected and uncorrected concentrations; however, the effect was stronger after applying residential seasonal correction factors. In the corrected dataset, measurements initiated during the warm season yielded significantly higher annual estimates than those initiated during the cold season (Mdn 49 vs. 31 Bq/m^3^, *p* < 0.001). By contrast, the corresponding measured, uncorrected concentrations showed nearly identical median values for warm- and cold-season starts (38 vs. 37 Bq/m^3^), indicating that the seasonal contrast became more pronounced only after correction (Figures 3a,b).

**Figure 3:**
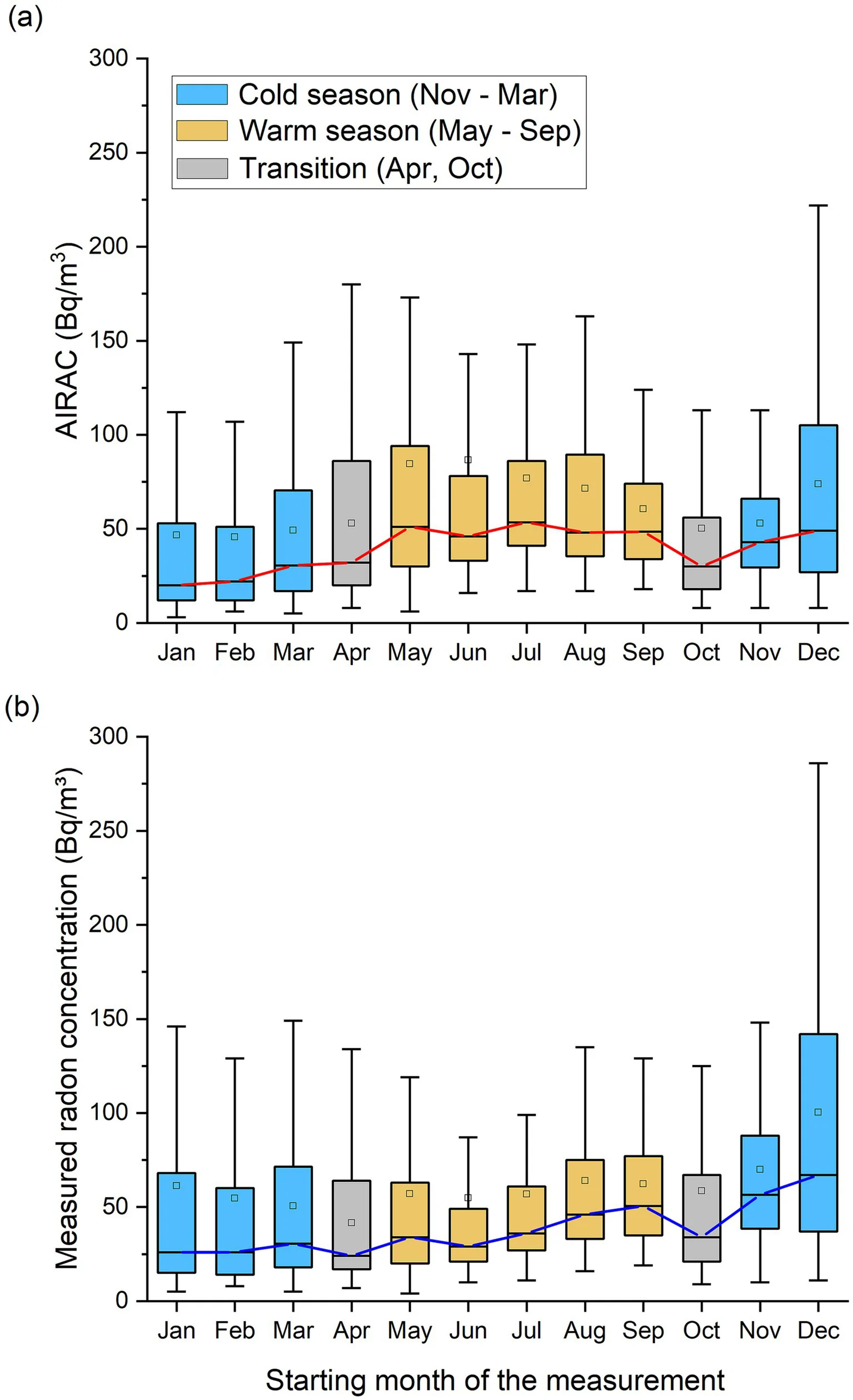
Monthly variation according to the starting month of measurement: comparison between seasonally corrected AIRAC **(a)** and uncorrected three-month radon concentrations **(b)**.

To further distinguish between measured radon variability and the effect introduced by seasonal correction, the same mixed-effects model was fitted separately to uncorrected three-month radon concentrations. In this model, floor level and medical facility type, remained significant predictors of log-transformed measured radon concentration (*p* < 0.001). However, starting month was not significant (*p* = 0.52). This indicates that the month-related pattern was much more pronounced after applying residential seasonal correction factors. Because different buildings and rooms were monitored in different months, this finding should not be interpreted as proof that the correction factors introduced bias at the level of individual measurements. Rather, it shows that the application of residential seasonal correction factors can substantially alter the starting-month pattern of annual radon estimates in this dataset.

## 4 Discussion

This study investigated the variation of indoor radon concentrations according to building level, measurement period, facility type and room function in 115 Romanian medical institutions. Of the 1,651 valid radon measurements included in the present study, only 26 values (1.6%) exceeded the reference level of 300 Bq/m^3^, with 18 of these exceedances occurring on the ground floor. This proportion is lower than that reported by Vaupotič and Kobal, who found radon concentrations above 300 Bq/m^3^ in 9 of the 201 rooms investigated in 26 Slovenian hospitals, corresponding to 4.5% of the monitored rooms (35). Comparable or even lower exceedance rates have been reported in Italian healthcare facilities. De Maria et al. observed exceedances of the reference level in 31 of the 3492 monitored rooms distributed across 30 medical buildings, corresponding to 0.9%, while the same percentage was reported by Vimercati et al. following radon monitoring in 401 rooms from 28 buildings of the University Hospital of Bari (31, 36). Similarly, in the largest public hospital in Southern Italy, Loffredo et al. reported concentrations above 300 Bq/m^3^ in only 3 of the 527 monitored rooms, corresponding to 0.6% (33). By contrast, a higher exceedance rate of approximately 5% was reported by Loffredo et al. in a survey of 607 rooms located at ground-floor level or in underground areas within 27 districts of a public healthcare company in Naples (39). In that study, the arithmetic mean radon concentration was 118 Bq/m^3^, exceeding the Italian national mean of 70 Bq/m^3^, calculated for residential buildings (40, 41).

For comparison with previous studies, measurement-level radon concentrations were considered; in the present dataset, the arithmetic mean was 61 Bq/m^3^, while the median and geometric mean were 40 Bq/m^3^ and 41 Bq/m^3^, respectively. As such, the arithmetic mean obtained in the present study, was substantially lower than the mean residential radon concentration of 173 Bq/m^3^ reported by Dicu et al. for the investigated regions of Romania (42). In fact, the percentage of measurements that exceeded the reference level in this study is one-tenth of those reported for dwellings (42, 43). Moreover, the mean value reported here for medical institutions was approximately three times lower than that obtained by Cucoş et al. for 198 educational buildings investigated in Romania (44).

Nevertheless, marked differences were observed among the types of medical facilities included in the survey. Higher median radon concentrations were recorded in radiology clinics (Mdn = 138 Bq/m^3^) and dental offices (Mdn = 93 Bq/m^3^), whereas lower values were observed in hospitals (Mdn = 55 Bq/m^3^) and medical clinics (Mdn = 47 Bq/m^3^). These differences were also supported by the linear mixed-effects model, in which facility type remained a significant predictor of log-transformed AIRAC even after accounting for floor level, starting month and the clustering of measurements within buildings.

This difference may be partially attributed to the regulatory and operational characteristics of the investigated facilities. In Romania, hospitals and medical clinics are subject to stricter technical and hygiene standards, including mandatory mechanical ventilation systems, minimum air exchange rates and controlled air renewal in functional areas. These provisions are detailed in the Technical Norm for Hospital Buildings (24) and in Order of the Ministry of Public Health No. 914/2006 concerning hygiene norms for hospital premises (25). Such requirements may contribute to more effective dilution and removal of indoor radon. In contrast, dental and radiology offices often operate in smaller spaces converted from residential or commercial use, where hospital-specific air-handling requirements may not apply and adequate mechanical ventilation may be absent. Notably, the highest radon concentrations in the present study were measured in a dental office (825 Bq/m^3^) and its associated radiology laboratory (1733 Bq/m^3^), both accommodated within the same residential building constructed before 1900. Although functionally used for medical services, this building was residential in origin and was not purpose-built as a healthcare facility.

Only one of the 514 measurements performed on upper floors, defined here as the first floor or higher, exceeded the reference level of 300 Bq/m^3^. By comparison, 25 exceedances were recorded among the 1137 measurements performed in basements, semi-basements and ground-floor rooms. Thus, the detection yield for reference level exceedances was approximately 2.2% on lower levels, compared with 0.2% on upper floors. A similar pattern was reported by De Maria et al. in the University Hospital of Bari, Southern Italy, where only one exceedance was identified among the 797 rooms monitored on the first floor, while no exceedances were recorded among the 861 rooms monitored on the second and third floors combined (585 and 276 rooms, respectively) (31).

These findings support the prioritization of lower building levels in radon monitoring strategies, but they should not be interpreted as evidence that upper-floor monitoring lacks regulatory value. Although upper-floor exceedances were rare in the present dataset, the identification of one exceedance among 514 upper-floor measurements confirms that such occurrences are possible. This issue is relevant in the Romanian regulatory context, where at least one detector per 250 m^2^ is recommended on upper floors in buildings with high occupancy, including medical facilities. Therefore, a risk-based monitoring strategy should prioritize basements, semi-basements and ground-floor rooms, while retaining targeted upper-floor monitoring where justified by building characteristics, occupancy, ventilation conditions, previous radon results or local radon potential. This is particularly important for dental offices or radiology clinics accommodated within spaces converted from residential or commercial use, where radon dynamics may differ from those of purpose-built healthcare facilities.

Another important discrepancy between the national radon measurement methodology and field implementation concerns the recommended timing of measurements. The national methodology adopts a conservative approach by recommending measurements during the cold season, when indoor radon concentrations are generally expected to be higher or closer to the annual means. However, in practice, measurements in medical buildings are often scheduled according to operational constraints, including the availability of institutional budgets, planned renovation works or administrative requirements. Consequently, although approximately half of the measurements in the present study covered the cold season, a substantial proportion also included the warm season. Interestingly, the median measured radon concentration during the cold season was almost identical to that observed during the warm season, 37 Bq/m^3^ and 38 Bq/m^3^, respectively, despite the fact that the monitored buildings were not the same.

Annual indoor radon activity concentrations (AIRAC) are commonly estimated by applying seasonal correction factors. However, temporal correction factors may vary substantially depending on building and measurement conditions, and this issue has previously been addressed using active environmental monitoring devices (45). In the Romanian methodology, these factors were developed from residential measurements, typically without explicit consideration of mechanical ventilation. Their application to medical buildings therefore requires caution, because healthcare facilities may differ from dwellings in ventilation regime, functional zoning and operational schedules. In the present dataset, warm-season measurements yielded substantially higher seasonally corrected AIRAC estimates than cold-season measurements (median 49 vs. 31 Bq/m^3^), whereas the corresponding uncorrected three-month concentrations were nearly identical (median 38 vs. 37 Bq/m^3^). The parallel mixed-effects models led to the same interpretation: starting month was not significant for uncorrected concentrations, but became marginally significant for seasonally corrected AIRAC. However, this result should not be interpreted as proof that the residential correction factors are inaccurate, because starting month determines the correction factor and the study was cross-sectional, with different buildings monitored in different months. Rather, the findings show that the application of residential seasonal correction factors amplified the warm–cold contrast and the apparent starting-month pattern of AIRAC estimates in this dataset. Direct validation of correction factors for medical buildings would require repeated seasonal measurements in the same rooms.

The present study has several limitations that should be acknowledged. First, the dataset was generated through regulatory monitoring rather than through a random or geographically balanced sampling design. Therefore, the investigated facilities should not be considered nationally representative of all Romanian medical buildings. The spatial distribution was uneven, with a large proportion of monitored facilities located in Cluj County, and this may limit the generalizability of the results. Although locality code was included as a random intercept in a sensitivity analysis to account partly for locality-level geographical variability, no direct geogenic radon potential indicators were available. Consequently, residual confounding by geology, county or local radon potential cannot be excluded. Second, the number of detectors varied substantially between facilities, reflecting differences in building size, functional complexity and regulatory monitoring requirements. This unequal sampling intensity may affect the precision of building-level mean AIRAC estimates and should be considered when interpreting comparisons between facility categories. In addition, facility type may be partly confounded with building age and location. For example, some dental or radiology offices were accommodated in buildings originally designed for residential or commercial use, whereas hospitals and larger clinics were more often purpose-built healthcare facilities. Therefore, differences attributed to facility type may also reflect differences in building age, construction characteristics, ventilation infrastructure or local setting. Third, important building- and operation-related factors, such as construction materials, type of ventilation system, duration of mechanical ventilation at room level, number of occupants, occupancy patterns and time spent indoors, were not available and therefore could not be included in the analysis. These parameters would also be necessary for a more refined estimation of the annual effective dose associated with occupational exposure to radon. In addition, repeated measurements in the same rooms or buildings over longer periods were not performed, which limited the possibility of directly assessing seasonal variability in medical buildings or validating seasonal correction factors for this category of facilities. Finally, although the dataset included a large number of measurements, some categories of medical facilities and room functions were represented by relatively small numbers of observations, which limits the statistical interpretation of subgroup comparisons.

Despite these limitations, the study provides several relevant contributions. To our knowledge, this is the first large-scale investigation of indoor radon concentrations in medical buildings in Romania. Moreover, the fact that measurements were carried out in 115 medical institutions indirectly reflects a growing, although still gradual, awareness of radon exposure and an increasing interest of public and private healthcare institutions in assessing the risks associated with this indoor radioactive pollutant. Finally, the results provide empirical evidence supporting the need for a more dynamic and context-sensitive regulatory approach. Such an approach should better account for the specific functional, operational and ventilation-related characteristics of medical buildings, with the aim of improving the estimation of AIRAC and ensuring a more realistic and effective implementation of radon monitoring requirements in the targeted buildings.

## 5 Conclusion

The present study provides the first large-scale assessment of indoor radon concentrations in Romanian medical buildings, based on 1,651 valid measurements performed in 115 medical institutions from 40 localities. Overall, radon concentrations were generally low, with only 1.6% of measurements exceeding the reference level of 300 Bq/m^3^. Exceedances were concentrated predominantly on lower building levels, including basements, semi-basements and ground-floor rooms, while only one exceedance was identified on upper floors. The results also showed substantial heterogeneity between types of medical facilities, with higher values observed in radiology clinics and dental offices than in hospitals and medical clinics. These findings indicate that floor level and facility type are important predictors of indoor radon variability in the surveyed medical buildings.

These findings underscore the need to reconsider certain methodological and regulatory assumptions regarding indoor radon monitoring in medical buildings. The low exceedance rate on upper floors supports prioritizing lower building levels, but does not justify omitting upper-floor monitoring, since rare exceedances remain relevant from a regulatory perspective. In addition, the stronger warm–cold contrast observed after applying residential seasonal correction factors indicates that seasonal adjustment can substantially affect annual radon estimates in healthcare facilities. A risk-based monitoring strategy that prioritizes lower levels and facility categories associated with higher concentrations, while retaining appropriate upper-floor coverage and further validating seasonal correction factors through repeated measurements, may provide a more realistic and efficient approach for medical buildings.

## Data Availability

The raw data supporting the conclusions of this article will be made available by the authors, without undue reservation.
